# Epigenetics in Alzheimer’s Disease: A Critical Overview

**DOI:** 10.3390/ijms25115970

**Published:** 2024-05-29

**Authors:** Chiara Villa, Romina Combi

**Affiliations:** School of Medicine and Surgery, University of Milano-Bicocca, 20900 Monza, Italy; chiara.villa@unimib.it

**Keywords:** epigenetics, Alzheimer’s disease, aging

## Abstract

Epigenetic modifications have been implicated in a number of complex diseases as well as being a hallmark of organismal aging. Several reports have indicated an involvement of these changes in Alzheimer’s disease (AD) risk and progression, most likely contributing to the dysregulation of AD-related gene expression measured by DNA methylation studies. Given that DNA methylation is tissue-specific and that AD is a brain disorder, the limitation of these studies is the ability to identify clinically useful biomarkers in a proxy tissue, reflective of the tissue of interest, that would be less invasive, more cost-effective, and easily obtainable. The age-related DNA methylation changes have also been used to develop different generations of epigenetic clocks devoted to measuring the aging in different tissues that sometimes suggests an age acceleration in AD patients. This review critically discusses epigenetic changes and aging measures as potential biomarkers for AD detection, prognosis, and progression. Given that epigenetic alterations are chemically reversible, treatments aiming at reversing these modifications will be also discussed as promising therapeutic strategies for AD.

## 1. Introduction

Aging, described as a progressive time-related deterioration of physiological integrity, is a continuous phenomenon and represents the strongest non-modifiable risk factor for neurodegenerative disorders, including Alzheimer’s disease (AD) [1]. A better understanding of the mechanistic links between the aging process and AD is thus crucial to determine when to deliver a preventive intervention based on brain aging measures [2]. As a result, most research is focused on estimating aging variations that may precede AD symptoms or development using biomarkers measuring an individual’s health, particularly those reflecting brain aging and biological aging [3].

Brain age is evaluated by determining neuroanatomical changes mainly using two estimators: the brainAGE (Brain Age Gap Estimation) method [4] and the one developed by Cole and collaborators [5]. Brain age may be influenced by some aging-related structural brain changes, such as atrophy, loss of white and grey matter, and disruptions to functional connectivity with increased grey matter atrophy. Estimating brain age successfully differentiates and predicts conversion to AD in subjects with mild cognitive impairment (MCI), the prodromal stage of AD [6]. An acceleration of brain aging was found in individuals who converted to AD within three years and those with pre-clinical AD [7]. 

At the cellular and molecular levels, biological age can be estimated using a variety of biomarkers that include epigenetic modifications, genomic instability, telomere attrition, the loss of proteostasis, deregulated nutrient-sensing, mitochondrial dysfunction, cellular and immunosenescence, stem cell exhaustion, and altered intercellular communication [8]. Among them, epigenetic alterations are one of the most important mechanisms driving the impaired cellular activities observed in aging and age-related diseases. By definition, epigenetics refers to meiotic and/or mitotic alterations that can regulate gene expression by modulating the chromatin structure or by affecting the binding of transcriptional machinery to DNA through reversible mechanisms without altering the DNA sequence [9]. DNA methylation (DNAm), histone modifications, and gene expression regulation mediated by micro(mi)RNAs and long non-coding RNAs (lncRNAs) represent the main epigenetic mechanisms [10]. So far, DNAm has been the most intensively researched one and is catalysed by DNA methyltransferase (DNMT), which adds a methyl group to the fifth position of a cytosine ring, resulting in the formation of 5-methylcytosine (5-mC). By altering chromosomal structure, DNA conformation, and DNA stability, DNAm regulates gene expression by recruiting proteins that inhibit genes or by impeding the interaction of transcription factor(s) with DNA [11]. Histone modifications are post-translational chemical changes that include acetylation, methylation, phosphorylation, ubiquitination, ADP-ribosylation, and SUMOylation. These alterations affect the structure of chromatin, resulting in either a euchromatinic state, which promotes gene transcription, or a heterochromatinic state, which is characterized by condensed chromatin and the suppression of gene expression [12]. In addition to DNAm and histone modifications, other machineries are involved in epigenetic regulation, such as miRNAs and lncRNAs. While miRNAs negatively regulate gene expression either by targeting mRNA degradation or by inhibiting protein translation, the mechanisms of action of lncRNAs are more complex, as they can interact with DNA, mRNA, protein, and miRNA, regulating gene expression at multiple levels, such as through chromatin remodelling, transcriptional activation, RNA processing, and mRNA translation [13]. Importantly, epigenetic mechanisms can be regulated and altered by environmental factors, such as age, diet, lifestyle, smoking, and stress [14]. With specific relevance to AD, epigenetic modifications, primarily DNAm, have been linked to disease onset and progression, most likely contributing to the dysregulation of AD-related genes [15]. Among them, some studies have found abnormal DNAm patterns in genes associated with synaptic plasticity, neuronal growth, inflammatory-immune responses, oxidative stress, memory impairment, and amyloid metabolism [16]. Over the last decade, several epigenetic clocks have been developed to estimate the biological aging of an individual based on age-associated changes in DNAm at specific CpG-sites scattered across the genome. Epigenetic age acceleration (EAA), defined as the discrepancy between chronological and DNAm age, has been associated with AD, with manifestations including deficits in episodic and working memory, as well as cortical thinning in the frontal, superior temporal, inferior parietal, medial, and occipital regions [17,18,19]. As a result, people with accelerated epigenetic age may develop AD symptoms earlier.

Herein, this review critically discusses epigenetic changes and aging measures as potential biomarkers for AD detection, prognosis, and progression. Given that epigenetic alterations are chemically reversible, treatments aiming at reversing these modifications will be also discussed as promising therapeutic strategies for AD.

## 2. The Heterogeneity of Epigenetic Clocks and Their Use in AD Evaluation

Epigenetic clocks are biological age estimators of an individual based on age-associated changes in DNAm at specific CpG-sites scattered across the genome. In recent years, several epigenetic clocks have been developed for many species. The first-generation epigenetic clocks, the Hannum clock [20] and the Horvath pan-tissue clock [21], were built using machine learning algorithms to predict chronological age based on DNAm levels at selected CpG loci with Illumina Infinium arrays. The Hannum clock is based on DNAm at 71 CpGs located near genes involved in age-related disorders and was originally created using peripheral blood samples [20]. Because this test is tissue-specific, it requires further calibration for its use in other tissues, making it impossible to compare the aging of different tissues. In contrast to the Hannum estimator, the Horvath pan-tissue clock has been constructed across 51 human tissues and cell types with the aim of developing a multi-tissue predictor for age. This clock encompasses 353 CpG markers, 193 of which positively correlated with age, and 160 of which showed a negative correlation [21]. The model exhibits excellent adaptability to various tissues and cell types, good accuracy, and a wide range of lifespans, as evidenced by data [22]. However, the Hannum and Horvath pan-tissue clocks show some limitations in their use. First, they systematically underestimate age in tissues from older people [23], probably due to the saturation at certain loci or confounding age-related effects. Moreover, these first clocks also faced criticism for excluding certain CpGs whose methylation patterns would indicate a divergence between epigenetic and chronological age [24]. As a result, second-generation clocks, such as PhenoAge and GrimAge, were recently developed to predict multi-disease age-associated disease and mortality by combining variables indicative of health status (e.g., plasma proteins, stress factors, and smoking habits) with chronological age. Among them, the highly accurate PhenoAge clock consists of 513 CpGs and is based on the measure of biological age incorporating ten clinical biomarkers predictive of mortality, including chronological age, glucose, albumin, creatinine, C-reactive protein levels, lymphocyte percentage, alkaline phosphatase, mean cell volume, red blood cell distribution width, and whole blood cell count [24]. Among people of the same chronological age, PhenoAge predicts the risk of all-cause mortality [24]. The other multifactorial clock GrimAge combines chronological age and sex with seven DNAm-based estimators of plasma proteins and one of smoking pack-years, that have previously been linked to morbidity or death [25]. The GrimAge clock compares biological and chronological age to measure the EAA, thus predicting lifespan and providing information on the risk of developing age-related disorders [25]. 

Later, Belsky and collaborators developed a third-generation blood-based epigenetic clock, called the Dunedin Pace of Aging methylation, (DunedinPoAm), a DNAm predictor of pace of aging [26]. DunedinPoAm compares longitudinal changes in 18 biomarkers of organ-system integrity (e.g., body mass index, leukocyte telomere length, and HDL cholesterol) over a 12-year observational period among individuals with the same chronological age to estimate the subject’s rate of aging. Individuals with higher DunedinPoAm scores had a higher likelihood of experiencing cognitive and physical losses by midlife, as well as accelerated facial aging [26]. Among older adults, higher DunedinPoAm scores were associated with an increased risk of disease and mortality [26,27]. Using the same Dunedin cohort with a longer follow-up period, the DNAm dataset was subsequently refined into a novel algorithm named DunedinPACE (Dunedin Pace of Aging Calculated from the Epigenome), showing a more precise measurement of the pace of aging than DunedinPoAm and a higher test–retest reliability [28]. The DunedinPACE measure was more predictive than previous generations of clocks when compared to clinical screening test scores, cognitive function evaluation, and clinical dementia diagnosis [29].

Importantly, these epigenetic clocks lose precision when applied to other tissues, despite having higher predicted accuracy within the particular tissues for which they were designed [30]. Indeed, they underestimate age in older samples and do not properly capture the distinctive aging changes in the brain because of its particular developmental patterns [31], great diversity of specialized neuronal and glial cell types [32,33], and peculiar methylation profile [34]. Hence, Shereby and collaborators developed the first human brain-specific epigenetic clock from samples of cortical tissue spanning the life course, namely DNAmClock_Cortical_, with the aim of identifying phenotypes associated with biological aging in the brain, such as dementia or neurodegenerative disorders [35]. When compared to existing epigenetic clocks designed for different tissues, DNAmClock_Cortical_ provides much better age predictions in human cortex samples across the lifespan, highlighting the need for brain-specific epigenetic clocks which may accurately reflect tissue-specific alterations related to biological aging [35]. Later, PCBrainAge was developed to capture meaningful heterogeneity of aging using DNAm data derived from multiple brain regions of 700 participants in the Religious Orders Study and Rush Memory and Aging Project [36]. This predictor demonstrates strong association with pathological and clinical phenotypes of AD as well as *APOE* ε4 carrier status. Furthermore, PCBrainAge shows cross-regional applicability in the brain. Unlike DNAmClock_Cortical_, considerable PCBrainAge acceleration was associated with an increased risk of dementia. This result is probably due to the reduced noise from CpGs and the enhanced resolution of the PCBrainAge clock project [36]. Given the evidence that the cerebellum ages slower than the rest of the human body according to the Horvath pan-tissue clock [37], a new brain cortex clock has recently been constructed, named the BrainCortexClock, which can accurately predict the DNAm age of samples from both cerebral and cerebellar cortices [38]. However, the majority of individuals included in this study are above the age of 60; future research should involve more young people to provide a more complete picture of age-related changes in the cerebellar methylome.

Due to the limited access of human brain tissues, two mouse brain region-specific epigenetic clocks were developed from hippocampus and cerebral cortex, both of which were significantly affected by AD and associated with cognitive decline [39]. This study revealed an EAA in both brain regions that was more prominent in the cortex than in the hippocampus. Age-dependent CpGs were shown to be enriched in genes involved in neuronal, neurodegenerative, aging, and developmental processes, according to genomic region enrichment analysis [39]. Although built for animals, these epigenetic clocks may be however very helpful in examining the timing of epigenetic age modification during the disease process, identifying risk factors for the accelerated aging associated with AD, and evaluating the effectiveness of potential interventions aimed at delaying the accumulation of epigenetic age. 

Despite the fact that these reported epigenetic clocks are extensively used and studied, they show some limitations. First, the majority of them were developed by individuals of European or Hispanic ancestry [20,21,24,25,28], although ethnic differences in DNAm patterns have been reported [24,40,41]. Apart from genetic confounding, environmental variations among populations also impact the physiological characteristics upon which these models are based. This is especially true when lifestyle factors are integrated, as is the case with the GrimAge clock [25], which captures the harmful consequences of smoking. Additionally, some epigenetic clocks, like DunedinPoAm, compare DNAm levels to physiological indicators of biological age (such as levels of cholesterol), which may be the result of confounding variables (such as obesity) rather than depicting aging itself [42]. Therefore, replication in different cohorts should continuously be pursued, and clocks adjusted when previously unmeasured confounding is discovered. Furthermore, the CpGs and Illumina profiles of the existing epigenetic clocks differ, resulting in significant variations in research and limitations in practical application [43]. Regarding brain-based epigenetic clocks, it is important to note that this tissue is obtained post-mortem, so it may not accurately reflect the mechanisms involved in AD development, but only provides insight into the final stages of AD-related processes [44]. Moreover, the analysis is restricted by the heterogeneity of brain tissue since it is well known that distinct cells exhibit unique methylation patterns [45].

## 3. Correlations between Epigenetic Alterations and Other Age-Related Hallmarks in AD

Accumulating evidence has demonstrated that epigenetic alterations can affect age-related mechanisms acting as accelerators of pathological aging [46]. Therefore, a better knowledge of the molecular processes underpinning the identified correlations could lead to the discovery of new avenues for reversing aging-associated processes, thus reducing the risk of AD development.

Several studies have demonstrated that telomere attrition, measured in terms of telomere length and telomere shortening rate, is directly correlated with cognitive decline and AD [47], although some authors reported contradictory results. Shorter telomeres in AD patients may be related to many epigenetic mechanisms, including DNAm in the promoter region of the catalytic subunit of the telomerase enzyme and the regulation mediated by several non-coding RNAs (ncRNAs), which are known to affect telomere dynamics [48]. In mice, miR-340-5p has been found to increase the length of telomeres, delay cell senescence, and relieve AD symptoms by lowering the expression of POT1, a key protein involved in the regulation of telomerase-mediated telomere elongation [49].

Aberrant proteostasis, the process to maintain the homeostasis of the proteome, is a feature of AD and contributes to neuronal stress, which in turn results in synapse loss and memory impairments [50]. Molecular chaperones and two proteolytic systems, the lysosome-autophagy and the ubiquitin–proteasome systems (UPS) play key roles in the maintenance of proteostasis [51]. In AD patients, DNA hypermethylation has been observed at the promoter region of the *FOXO3A* gene, encoding a protective transcriptional regulator involved in the maintenance of cell homeostasis from environmental stress by increasing autophagy [52]. 

Mitochondrial dysfunction plays an important role in AD pathogenesis, since mitochondria regulate both cellular metabolism and apoptosis [53]. A DNAm increase in the promoter of the *EOVL2* gene has been reported in early-stage AD cases compared to controls, resulting in increased endoplasmic reticulum stress and mitochondrial dysfunction [54]. Interestingly, the same authors found a correlation between *ELOVL2* methylation levels and p-tau protein deposits in the human hippocampal regions, raising the possibility that this gene plays a role in the development and progression of AD [54]. In addition to nuclear DNA, the expression of genes in the mitochondria is similarly regulated by epi-genetic mechanisms, mainly DNAm [55]. In the majority of AD animal models and human samples, a demethylation of the D-loop region, which regulates the transcription and regulation of mitochondrial DNA (mtDNA), has been found, while the genes encoding 12S rRNA, CYTB, and COX II were hypermethylated with decreased mtDNA copy numbers. It is possible to speculate that D-loop demethylation may compensate for the hypermethylation of 12S rRNA, CYTB, and COX II-encoded genes, although it is still unclear whether the mtDNA methylation alterations are the cause or the effect of AD [56]. 

In AD development, a crucial role is also played by immunosenescence, the age-associated deterioration of the immune system which is characterized by a decline in overall immune function and higher levels of inflammatory markers [57]. It has been reported that the inflammatory response of microglia and astrocytes is stimulated by aberrant epigenetic modifications of the gene promoters of cytokines like TNF-α, IL-1β, and IL-6. This leads to the formation of pathogenic Aβ deposits and neurofibrillary tangles, which ultimately cause the development and exacerbation of AD [58]. Moreover, a study performed on familial AD patients revealed hypermethylation in the promoter regions of *KLF14*, encoding for a protein involved in immune cell differentiation [59].

## 4. Epigenetic Modifications as Possible Diagnostic/Prognostic Biomarkers for AD

Among the currently used strategies in the diagnosis of possible/probable AD, the identification and use of biomarkers has been challenging in recent years. Several biomarkers have been detected in blood and cerebrospinal fluid (CSF) and through the availability of imaging techniques [60,61]. However, not all known biomarkers are suitable for use in clinical practice due to their level of invasiveness and, more strictly, to their high costs, as well as the need for specialists able to interpret the results [62,63]. It is therefore urgent to find less invasive, more cost-effective, and easily obtainable biomarkers, and possibly biomarkers sensitive and specific for predicting disease onset and progression. 

Recent focus has been on the ability of epigenetic biomarkers to predict age-related neurodegeneration and also AD. It was in fact suggested by Wang and colleagues [64] that the onset of AD represents the crossing of a particular threshold of epigenetic deregulation with late-onset AD (LOAD) being the extreme form of aging with epigenetic alterations in the brain. The epigenetic role in AD has been studied with different approaches (e.g., studies on animal models, mainly rats and cynomolgus monkeys) showing that the environment condition in early age might impact the disease pathology later in life through an alteration of the amyloidogenic Aβ levels of expression [65] and studies on plasma levels of homocysteine (Hcy) demonstrating an inverse linear relationship between them and the cognitive function in older individuals [66,67].

Several studies concentrated their attention specifically on global and gene-specific DNAm in AD. 

Since the publication of a twin study in 2009 [68], it has been found several times that monozygotic twins with phenotypic discordance (AD vs healthy) could show differences in global DNAm. Subsequent studies demonstrated brain region differences in methylation patterns, highlighting the difficulty in drawing a clear picture of methylation in such an heterogeneous disease as AD [44]. There are numerous genes associated with AD progression that are epigenetically regulated.

Moreover, a reduced condensation of constitutive heterochromatin regions on chromosomes 1, 9, and 16 was reported in patients vs controls [69].

Another strategy used to simplify the study consists in studying gene specific methylation patterns of known AD loci starting from the *APOE* whose Ɛ4 allele is considered the strongest genetic risk factor for the disease. Although different papers reported a hypermethylation of the *APOE* promoter in AD patients [70,71], no significant differences were found in other studies [72]. In particular, DNAm at two *APOE* CpG sites (already known to undergo age-dependent changes) was related to cholesterol levels but not to cognitive decline and AD, supporting that there is no evidence yet for considering *APOE* methylation as a biomarker for predicting AD [72]. However, longitudinal studies with methylation profiles have to be done to confirm a link between AD and APOE epigenetics.

The methylation levels of other AD loci (*APP*, *MAPT*, *PSEN1*, *BACE1*, and *BDNF*) have also been studied, resulting in non-concordant reports [73,74,75,76,77,78]. This could be due to differences in studied cohorts as well as differences in the biological sample used for the analysis (blood or post-mortem tissue from different brain regions). The promoter of the *MTHFR* gene and *ANKYRIN* were found to be hypermethylated both in post-mortem brain tissues and/or in peripheral lymphocytes [64,79].

In general, altered DNAm correlates with increased Aβ levels, tau neurofibrillary tangle density, increased neuritic plaques, and increased cortical pathology [17,24,64,79,80,81,82].

Few papers evaluated the existence of differences in specific gene methylation patterns in healthy controls vs early or later stages of AD. In particular, an increasing hypomethylation state was reported for the IL-1β promoter and IL-6 with disease progression [83].

Recent human large-scale epigenome studies focusing on histone modifications in AD reported a loss of some histone marks and a gain of other marks, demonstrating the complex dynamics of histone modifications, particularly in LOAD [for a survey of reported histone modification see [84].

A strong association has been reported between AD risk factors (i.e., body mass index, socioeconomic status, total cholesterol to HDL cholesterol ratios, high blood pressure, and smoking behaviour) and both age-acceleration [85] and epigenetic aging [86]. The use of multiple epigenetic clocks based on DNAm, such as Hannum, Horvath, PhenoAge, and DNAmClock_Cortical_, showed the existence of a relationship between DNAm age, AD, and Aβ load that was stronger using the cortical one [87]. Moreover, an age-acceleration was seen in murine models of AD [39,88].

A limited number of studies using different biological samples (either blood or brain samples) and different epigenetic clocks have investigated the association between DNAm age and AD onset. Some papers reported an association between an EAA and the disease, while several others did not obtain the same results.

All of these discordant results could be explained taking into account the limitations of currently reported studies. In fact, the starting biological sample chosen for analysis is of great. It is well known that methylation levels vary among tissues and therefore, results obtained using brain tissues are not comparable with those obtained using peripheral tissue (e.g., blood). Moreover, even using the target organ tissue (i.e., brain tissue for AD) the researcher is forced to use a heterogeneous population of cells and this could be reflected in methylation profiles. Additionally, generally, these studies rely on post-mortem brain and this is challenging for data interpretation due to the fact that altered methylation patterns could possibly be caused by the cause of death and other environmental factors [89].

The utility of studying blood has been questioned because it is not a real mirror of changes found in the brain. However, a good peripheral biomarker should not have the function of mirroring the brain but it could represent the peripheral responses to those changes. Then, further investigations are needed focusing on epigenetic changes in blood DNA before and following the onset of the disease and in different stages of its progression with the purpose of identifying prognostic/diagnostic biomarkers. 

The expression of miRNAs in the brain as well as in the biofluid has been reported to be altered in AD suggesting that these molecules could be used as biomarkers or therapeutic targets (Table 1) [90,91]. For example, miRNA-29a/b-1 [90,92,93] and miRNA-132 [94] were reported to be decreased while miRNA-34c increased [95,96]. Similarly, lncRNAs may be used as potential diagnostic and prognostic biomarkers [97]. For instance, BACE1-AS was found to be upregulated in the plasma of AD patients compared to healthy controls, suggesting its possible role as biomarker for AD diagnosis [98]. Moreover, another study that subdivided the patient group into pre-AD and full-AD based on an MMSE evaluation of the disease’s progression found that BACE1-AS plasma levels were lower in pre-AD individuals than those of the full-AD patients and healthy subjects, strengthening its potency as a predictive biomarker [99].

## 5. Therapies

Actually, the currently approved treatments for AD consist in the administration of acetylcholinesterase inhibitors (donepezil, galantamine, and rivastigmine) and antagonists of the glutamate NMDA receptor. However, novel approaches are needed to slow, delay, or even reverse AD clinical pathology. 

A possible new approach could consist in improving the chaperone-mediated autophagy (CMA), which is normally impaired in AD cells. CMA selectively recognises proteins containing a KFERQ motif, favouring their translocation to the lysosomes and then their degradation. All relevant AD proteins (APP, tau, α-synuclein, and LRKK2) contain this motif but the CMA in patients is impaired [118]. The administration of CMA activators (CA77.1, metformin, trehalose, lactulose, and PRO-Br) showed efficacy in AD mouse models [119,120,121].

Considering the reported DNAm changes in AD patients, a possible future treatment could rely on controlling this feature.

Several kinds of epigenetic approaches could be considered. The first one consists in a partial reprogramming of cells focused on restoring them to a more youthful state by removing epigenetic marks using a combination of three transcription factors (Oct4, Sox2, and Klf4) [122]. However, the delivery of gene therapies will be challenging because multiple factors must be addressed in the brain without expression in non-target organs and the therapies must have a sufficient penetration in the brain to induce expression for therapeutic efficacy and safety. Delivery systems have to be developed for this scope.

Another target mechanism for an epigenetic therapy for AD could be histone acetylation. Several changes in this process were reported in AD: a strong reduction in cerebral cortex levels of histone H4 acetylated at the 16th lysine residue [123] and an increased level of histone deacetylase 2 (HDAC2) in the hippocampus of patients and AD mouse models [124]. The use of HDAC2-inhibiting drugs (e.g., CI-994 or sulforaphane) could then be useful in AD therapy as demonstrated by studies in animals [125]. The advantage of the use of HDAC inhibitors is that a group of them (M344, CM-414, and RGFP-966) affect multiple genes involved in AD and this is particularly important due to the heterogeneity of the disease. Unfortunately, these drugs are wide spectrum compounds and could have adverse effects. A newly designed HDAC inhibitor (HDACi W2) demonstrated, in mouse models, its efficacy in reducing Aβ production as well as decreasing the phosphorylation of the tau protein [126]. Valproate (a class I HDAC inhibitor) is also considered as a possible treatment for AD as well as vitamin B3 and vorinostat. Clinical trials are ongoing for these drugs.

New methodological advances for AD treatment have developed short and synthetic antisense oligonucleotides that recognize target mRNA for posttranscriptional regulation to correct protein expression errors [127]. Moreover, a clinical trial is ongoing for the use of Gemfibrozil, a drug that modifies miR-107 levels, which in turn, regulates BACE1 expression.

An emerging epigenome therapeutic strategy for preventing or delaying the onset of AD is provided by CRISPR/Cas9 editing technology based on the use of an engineered nuclease-deficient version Cas9 protein (dCas9), whose only function is to bind to target-specific loci without cleavage [128]. For instance, dCas9 can be fused with different effector domains to generate CRISPR activation (CRISPRa) or CRISPR interference (CRISPRi) that target specific promoter regions, enhancing or inhibiting gene expression, respectively [129]. Some authors successfully developed an in vivo Cas9 activator nanocomplex for overexpressing Adam10, which is related to α-secretase activity. Results demonstrated that the Adam10 activator alleviates the neurotoxic deposition of Aβ and improves cognitive deficits [130]. Recently, targeted DNAm of the APP promoter mediated by dCas9 fused with DNA methyltransferase 3 (Dnmt3) has been shown to reduce Aβ levels and improve cognitive and behavioural impairments in a mouse model of AD [131]. In addition, nonspecific epigenetic therapies could be considered, such as blood plasma therapy from young subjects, cognitive stimulation, physical exercise, and dietary interventions. There are several concerns in the use of epigenetic therapy for AD: epigenetic changes are complex and therapies could have side effects; not all epigenetic changes can be reversed; and different regions of one gene can have antagonistic epigenetic changes.

## 6. Conclusions and Future Directions

This review examines the significance and utility of epigenetics in studying, preventing, and treating AD. Although to date some promising clinical trials are ongoing to evaluate new epigenetics therapies for AD using different approaches, no sufficient support is reported indicating that epigenetic aging measured in blood or different tissues and using existing epigenetic clocks could be a biomarker of risk for the disease. Additional studies are required to determine whether new epigenetic clocks developed to specifically predict age-related disease could be more accurate biomarkers and to understand the extent to which aging is a causal factor of AD risk. The use of other epigenetic biomarkers such as DNAm changes in AD needs additional studies due to the limited predictive value and the heterogeneity of results using different tissues and cohorts.

## Figures and Tables

**Table 1:** A summary of the most dysregulated miRNAs as potential biomarkers for AD.

MiRNA	Expression	Key Target (s)	Sample/Tissue	References
miR-9	↓	*BACE1*	serum	[100]
miR-15b	↓	*BACE1*	brain	[101]
miR-16	↓	*APP*	frontal cortex, serum	[102]
miR-23a	↑	*ADAM10*	serum	[103]
miR-26a	↓	*GSK3B*	serum	[104,105]
miR-26b	↑	*Rb1*	serum, whole blood, CSF	[106,107,108]
miR-29a	↓	*BACE1*	brain, serum	[90,92]
miR-29b	↓	*BACE1*	blood	[93]
miR-34c	↑	*SIRT1*	serum, blood, mononuclear cells	[95,96]
miR-107	↓	*BACE1, ADAM10*	plasma, whole blood	[109,110]
miR-124	↓	*BACE1*	brain	[111]
miR-125b	↑	*NF-kB*	serum	[112]
miR-132	↓	*MMP9*	serum	[94]
miR-146a	↓	*NF-kB, IRAK-1*	plasma, CSF	[113]
miR-181a	↓	*GRIA2*	serum	[114]
miR-181c	↓	*SIRT1*	serum	[92]
miR-206	↑	*BDNF*	plasma	[115,116]
miR-501-3p	↓	*GRIA1*	serum	[117]

## Data Availability

Not applicable.

## References

[1] Guerreiro R., Bras J. (2015). The age factor in Alzheimer’s disease. Genome Med..

[2] Franke K., Gaser C. (2019). Ten Years of BrainAGE as a Neuroimaging Biomarker of Brain Aging: What Insights Have We Gained?. Front. Neurol..

[3] Liang W.S., Goetz L.H., Schork N.J. (2022). Assessing brain and biological aging trajectories associated with Alzheimer’s disease. Front. Neurosci..

[4] Franke K., Ziegler G., Klöppel S., Gaser C. (2010). Estimating the age of healthy subjects from T1-weighted MRI scans using kernel methods: Exploring the influence of various parameters. Neuroimage.

[5] Cole J.H., Leech R., Sharp D.J. (2015). Prediction of brain age suggests accelerated atrophy after traumatic brain injury. Ann. Neurol..

[6] Gaser C., Franke K., Klöppel S., Koutsouleris N., Sauer H. (2013). BrainAGE in Mild Cognitive Impaired Patients: Predicting the Conversion to Alzheimer’s Disease. PLoS ONE.

[7] Gonneaud J., Baria A.T., Pichet Binette A., Gordon B.A., Chhatwal J.P., Cruchaga C., Jucker M., Levin J., Salloway S., Farlow M. (2021). Accelerated functional brain aging in pre-clinical familial Alzheimer’s disease. Nat. Commun..

[8] López-Otín C., Blasco M.A., Partridge L., Serrano M., Kroemer G. (2023). Hallmarks of aging: An expanding universe. Cell.

[9] Wu C., Morris J.R. (2001). Genes, genetics, and epigenetics: A correspondence. Science.

[10] Liu X., Jiao B., Shen L. (2018). The Epigenetics of Alzheimer’s Disease: Factors and Therapeutic Implications. Front. Genet..

[11] Christopher M.A., Kyle S.M., Katz D.J. (2017). Neuroepigenetic mechanisms in disease. Epigenetics Chromatin.

[12] Bano D., Salomoni P., Ehninger D., Nicotera P. (2021). The histone code in dementia: Transcriptional and chromatin plasticity fades away. Curr. Opin. Pharmacol..

[13] Zhang X., Wang W., Zhu W., Dong J., Cheng Y., Yin Z., Shen F. (2019). Mechanisms and Functions of Long Non-Coding RNAs at Multiple Regulatory Levels. Int. J. Mol. Sci..

[14] Zoghbi H.Y., Beaudet A.L. (2016). Epigenetics and Human Disease. Cold Spring Harb. Perspect. Biol..

[15] Qureshi I.A., Mehler M.F. (2018). Epigenetic mechanisms underlying nervous system diseases. Handb. Clin. Neurol..

[16] Gao X., Chen Q., Yao H., Tan J., Liu Z., Zhou Y., Zou Z. (2022). Epigenetics in Alzheimer’s Disease. Front. Aging Neurosci..

[17] Levine M.E., Lu A.T., Bennett D.A., Horvath S. (2015). Epigenetic age of the pre-frontal cortex is associated with neuritic plaques, amyloid load, and Alzheimer’s disease related cognitive functioning. Aging.

[18] Pellegrini C., Pirazzini C., Sala C., Sambati L., Yusipov I., Kalyakulina A., Ravaioli F., Kwiatkowska K.M., Durso D.F., Ivanchenko M. (2021). A Meta-Analysis of Brain DNA Methylation Across Sex, Age, and Alzheimer’s Disease Points for Accelerated Epigenetic Aging in Neurodegeneration. Front. Aging Neurosci..

[19] Proskovec A.L., Rezich M.T., O’Neill J., Morsey B., Wang T., Ideker T., Swindells S., Fox H.S., Wilson T.W. (2020). Association of Epigenetic Metrics of Biological Age with Cortical Thickness. JAMA Netw. Open.

[20] Hannum G., Guinney J., Zhao L., Zhang L., Hughes G., Sadda S., Klotzle B., Bibikova M., Fan J.B., Gao Y. (2013). Genome-wide methylation profiles reveal quantitative views of human aging rates. Mol. Cell.

[21] Horvath S. (2013). DNA methylation age of human tissues and cell types. Genome Biol..

[22] Horvath S., Raj K. (2018). DNA methylation-based biomarkers and the epigenetic clock theory of ageing. Nat. Rev. Genet..

[23] El Khoury L.Y., Gorrie-Stone T., Smart M., Hughes A., Bao Y., Andrayas A., Burrage J., Hannon E., Kumari M., Mill J. (2019). Systematic underestimation of the epigenetic clock and age acceleration in older subjects. Genome Biol..

[24] Levine M.E., Lu A.T., Quach A., Chen B.H., Assimes T.L., Bandinelli S., Hou L., Baccarelli A.A., Stewart J.D., Li Y. (2018). An epigenetic biomarker of aging for lifespan and healthspan. Aging.

[25] Lu A.T., Quach A., Wilson J.G., Reiner A.P., Aviv A., Raj K., Hou L., Baccarelli A.A., Li Y., Stewart J.D. (2019). DNA methylation GrimAge strongly predicts lifespan and healthspan. Aging.

[26] Belsky D.W., Caspi A., Arseneault L., Baccarelli A., Corcoran D.L., Gao X., Hannon E., Harrington H.L., Rasmussen L.J., Houts R. (2020). Quantification of the pace of biological aging in humans through a blood test, the DunedinPoAm DNA methylation algorithm. eLife.

[27] Graf G.H., Crowe C.L., Kothari M., Kwon D., Manly J.J., Turney I.C., Valeri L., Belsky D.W. (2022). Testing Black-White Disparities in Biological Aging among Older Adults in the United States: Analysis of DNA-Methylation and Blood-Chemistry Methods. Am. J. Epidemiol..

[28] Belsky D.W., Caspi A., Corcoran D.L., Sugden K., Poulton R., Arseneault L., Baccarelli A., Chamarti K., Gao X., Hannon E. (2022). DunedinPACE, a DNA methylation biomarker of the pace of aging. eLife.

[29] Sugden K., Caspi A., Elliott M.L., Bourassa K.J., Chamarti K., Corcoran D.L., Hariri A.R., Houts R.M., Kothari M., Kritchevsky S. (2022). Association of Pace of Aging Measured by Blood-Based DNA Methylation with Age-Related Cognitive Impairment and Dementia. Neurology.

[30] Zhang Q., Vallerga C.L., Walker R.M., Lin T., Henders A.K., Montgomery G.W., He J., Fan D., Fowdar J., Kennedy M. (2019). Improved precision of epigenetic clock estimates across tissues and its implication for biological ageing. Genome Med..

[31] Stiles J., Jernigan T.L. (2010). The basics of brain development. Neuropsychol. Rev..

[32] Zeng H., Sanes J.R. (2017). Neuronal cell-type classification: Challenges, opportunities and the path forward. Nat. Rev. Neurosci..

[33] Mizeracka K., Heiman M.G. (2015). The many glia of a tiny nematode: Studying glial diversity using Caenorhabditis elegans. Wiley Interdiscip. Rev. Dev. Biol..

[34] Spiers H., Hannon E., Schalkwyk L.C., Bray N.J., Mill J. (2017). 5-hydroxymethylcytosine is highly dynamic across human fetal brain development. BMC Genom..

[35] Shireby G.L., Davies J.P., Francis P.T., Burrage J., Walker E.M., Neilson G.W.A., Dahir A., Thomas A.J., Love S., Smith R.G. (2020). Recalibrating the epigenetic clock: Implications for assessing biological age in the human cortex. Brain.

[36] Thrush K.L., Bennett D.A., Gaiteri C., Horvath S., Dyck C.H.V., Higgins-Chen A.T., Levine M.E. (2022). Aging the brain: Multi-region methylation principal component based clock in the context of Alzheimer’s disease. Aging.

[37] Horvath S., Mah V., Lu A.T., Woo J.S., Choi O.W., Jasinska A.J., Riancho J.A., Tung S., Coles N.S., Braun J. (2015). The cerebellum ages slowly according to the epigenetic clock. Aging.

[38] Wang Y., Grant O.A., Zhai X., McDonald-Maier K.D., Schalkwyk L.C. (2024). Insights into ageing rates comparison across tissues from recalibrating cerebellum DNA methylation clock. Geroscience.

[39] Coninx E., Chew Y.C., Yang X., Guo W., Coolkens A., Baatout S., Moons L., Verslegers M., Quintens R. (2020). Hippocampal and cortical tissue-specific epigenetic clocks indicate an increased epigenetic age in a mouse model for Alzheimer’s disease. Aging.

[40] Tajuddin S.M., Hernandez D.G., Chen B.H., Noren Hooten N., Mode N.A., Nalls M.A., Singleton A.B., Ejiogu N., Chitrala K.N., Zonderman A.B. (2019). Novel age-associated DNA methylation changes and epigenetic age acceleration in middle-aged African Americans and whites. Clin. Epigenetics.

[41] Liu Z., Chen B.H., Assimes T.L., Ferrucci L., Horvath S., Levine M.E. (2019). The role of epigenetic aging in education and racial/ethnic mortality disparities among older U.S. Women. Psychoneuroendocrinology.

[42] Bergsma T., Rogaeva E. (2020). DNA Methylation Clocks and Their Predictive Capacity for Aging Phenotypes and Healthspan. Neurosci. Insights.

[43] Margiotti K., Monaco F., Fabiani M., Mesoraca A., Giorlandino C. (2023). Epigenetic Clocks: In Aging-Related and Complex Diseases. Cytogenet. Genome Res..

[44] Milicic L., Porter T., Vacher M., Laws S.M. (2023). Utility of DNA Methylation as a Biomarker in Aging and Alzheimer’s Disease. J. Alzheimer’s Dis. Rep..

[45] Hannon E., Lunnon K., Schalkwyk L., Mill J. (2015). Interindividual methylomic variation across blood, cortex, and cerebellum: Implications for epigenetic studies of neurological and neuropsychiatric phenotypes. Epigenetics.

[46] Ben-Shlomo Y., Cooper R., Kuh D. (2016). The last two decades of life course epidemiology, and its relevance for research on ageing. Int. J. Epidemiol..

[47] Guo Y., Yu H. (2019). Leukocyte Telomere Length Shortening and Alzheimer’s Disease Etiology. J. Alzheimer’s Dis..

[48] Koziel J.E., Fox M.J., Steding C.E., Sprouse A.A., Herbert B.S. (2011). Medical genetics and epigenetics of telomerase. J. Cell. Mol. Med..

[49] Li X., Zhang J., Yang Y., Wu Q., Ning H. (2021). MicroRNA-340-5p increases telomere length by targeting telomere protein POT1 to improve Alzheimer’s disease in mice. Cell Biol. Int..

[50] Cozachenco D., Ribeiro F.C., Ferreira S.T. (2023). Defective proteostasis in Alzheimer’s disease. Ageing Res. Rev..

[51] Kaushik S., Cuervo A.M. (2015). Proteostasis and aging. Nat. Med..

[52] Pradhan R., Yadav S.K., Prem N.N., Bhagel V., Pathak M., Shekhar S., Gaikwad S., Dwivedi S.N., Bal C.S., Dey A.B. (2020). Serum FOXO3A: A ray of hope for early diagnosis of Alzheimer’s disease. Mech. Ageing Dev..

[53] Wang W., Zhao F., Ma X., Perry G., Zhu X. (2020). Mitochondria dysfunction in the pathogenesis of Alzheimer’s disease: Recent advances. Mol. Neurodegener..

[54] Blanco-Luquin I., Acha B., Urdánoz-Casado A., Sánchez-Ruiz De Gordoa J., Vicuña-Urriza J., Roldán M., Labarga A., Zelaya M.V., Cabello C., Méndez-López I. (2020). Early epigenetic changes of Alzheimer’s disease in the human hippocampus. Epigenetics.

[55] Sharma N., Pasala M.S., Prakash A. (2019). Mitochondrial DNA: Epigenetics and environment. Environ. Mol. Mutagen..

[56] Song Y., Zhu X.Y., Zhang X.M., Xiong H. (2022). Targeted Mitochondrial Epigenetics: A New Direction in Alzheimer’s Disease Treatment. Int. J. Mol. Sci..

[57] Chen H.Y., Zhao Y., Xie Y.Z. (2023). Immunosenescence of brain accelerates Alzheimer’s disease progression. Rev. Neurosci..

[58] Ma Y., Wang W., Liu S., Qiao X., Xing Y., Zhou Q., Zhang Z. (2023). Epigenetic Regulation of Neuroinflammation in Alzheimer’s Disease. Cells.

[59] Wezyk M., Spólnicka M., Pośpiech E., Pepłońska B., Zbieć-Piekarska R., Ilkowski J., Styczyńska M., Barczak A., Zboch M., Filipek-Gliszczynska A. (2018). Hypermethylation of TRIM59 and KLF14 Influences Cell Death Signaling in Familial Alzheimer’s Disease. Oxid. Med. Cell. Longev..

[60] Villa C., Lavitrano M., Salvatore E., Combi R. (2020). Molecular and Imaging Biomarkers in Alzheimer’s Disease: A Focus on Recent Insights. J. Pers. Med..

[61] Villa C., Stoccoro A. (2022). Epigenetic Peripheral Biomarkers for Early Diagnosis of Alzheimer’s Disease. Genes.

[62] Dubois B., Hampel H., Feldman H.H., Scheltens P., Aisen P., Andrieu S., Bakardjian H., Benali H., Bertram L., Blennow K. (2016). Preclinical Alzheimer’s disease: Definition, natural history, and diagnostic criteria. Alzheimer’s Dement..

[63] Villa C. (2020). Biomarkers for Alzheimer’s Disease: Where Do We Stand and Where Are We Going?. J. Pers. Med..

[64] Wang S.C., Oelze B., Schumacher A. (2008). Age-specific epigenetic drift in late-onset Alzheimer’s disease. PLoS ONE.

[65] Basha M.R., Wei W., Bakheet S.A., Benitez N., Siddiqi H.K., Ge Y.W., Lahiri D.K., Zawia N.H. (2005). The fetal basis of amyloidogenesis: Exposure to lead and latent overexpression of amyloid precursor protein and beta-amyloid in the aging brain. J. Neurosci..

[66] Hooshmand B., Solomon A., Kåreholt I., Leiviskä J., Rusanen M., Ahtiluoto S., Winblad B., Laatikainen T., Soininen H., Kivipelto M. (2010). Homocysteine and holotranscobalamin and the risk of Alzheimer disease: A longitudinal study. Neurology.

[67] Román G.C., Mancera-Páez O., Bernal C. (2019). Epigenetic Factors in Late-Onset Alzheimer’s Disease: MTHFR and CTH Gene Polymorphisms, Metabolic Transsulfuration and Methylation Pathways, and B Vitamins. Int. J. Mol. Sci..

[68] Mastroeni D., McKee A., Grover A., Rogers J., Coleman P.D. (2009). Epigenetic differences in cortical neurons from a pair of monozygotic twins discordant for Alzheimer’s disease. PLoS ONE.

[69] Payão S.L., Smith M.D., Bertolucci P.H. (1998). Differential chromosome sensitivity to 5-azacytidine in Alzheimer’s disease. Gerontology.

[70] Wang C., Yu J.T., Wang H.F., Jiang T., Tan C.C., Meng X.F., Soares H.D., Tan L. (2014). Meta-analysis of peripheral blood apolipoprotein E levels in Alzheimer’s disease. PLoS ONE.

[71] Karlsson I.K., Ploner A., Wang Y., Gatz M., Pedersen N.L., Hägg S. (2018). Apolipoprotein E DNA methylation and late-life disease. Int. J. Epidemiol..

[72] Mur J., McCartney D.L., Walker R.M., Campbell A., Bermingham M.L., Morris S.W., Porteous D.J., McIntosh A.M., Deary I.J., Evans K.L. (2020). DNA methylation in APOE: The relationship with Alzheimer’s and with cardiovascular health. Alzheimer’s Dement..

[73] Iwata A., Nagata K., Hatsuta H., Takuma H., Bundo M., Iwamoto K., Tamaoka A., Murayama S., Saido T., Tsuji S. (2014). Altered CpG methylation in sporadic Alzheimer’s disease is associated with APP and MAPT dysregulation. Hum. Mol. Genet..

[74] Fransquet P.D., Lacaze P., Saffery R., Phung J., Parker E., Shah R.C., Murray A., Woods R.L., Ryan J. (2020). DNA methylation analysis of candidate genes associated with dementia in peripheral blood. Epigenomics.

[75] Barrachina M., Ferrer I. (2009). DNA methylation of Alzheimer disease and tauopathy-related genes in postmortem brain. J. Neuropathol. Exp. Neurol..

[76] Tannorella P., Stoccoro A., Tognoni G., Petrozzi L., Salluzzo M.G., Ragalmuto A., Siciliano G., Haslberger A., Bosco P., Bonuccelli U. (2015). Methylation analysis of multiple genes in blood DNA of Alzheimer’s disease and healthy individuals. Neurosci. Lett..

[77] Nagata T., Kobayashi N., Ishii J., Shinagawa S., Nakayama R., Shibata N., Kuerban B., Ohnuma T., Kondo K., Arai H. (2015). Association between DNA Methylation of the BDNF Promoter Region and Clinical Presentation in Alzheimer’s Disease. Dement. Geriatr. Cogn. Dis. Extra.

[78] Chang L., Wang Y., Ji H., Dai D., Xu X., Jiang D., Hong Q., Ye H., Zhang X., Zhou X. (2014). Elevation of peripheral BDNF promoter methylation links to the risk of Alzheimer’s disease. PLoS ONE.

[79] Lunnon K., Smith R., Hannon E., De Jager P.L., Srivastava G., Volta M., Troakes C., Al-Sarraj S., Burrage J., Macdonald R. (2014). Methylomic profiling implicates cortical deregulation of ANK1 in Alzheimer’s disease. Nat. Neurosci..

[80] Bakulski K.M., Dolinoy D.C., Sartor M.A., Paulson H.L., Konen J.R., Lieberman A.P., Albin R.L., Hu H., Rozek L.S. (2012). Genome-wide DNA methylation differences between late-onset Alzheimer’s disease and cognitively normal controls in human frontal cortex. J. Alzheimer’s Dis..

[81] Sanchez-Mut J.V., Aso E., Panayotis N., Lott I., Dierssen M., Rabano A., Urdinguio R.G., Fernandez A.F., Astudillo A., Martin-Subero J.I. (2013). DNA methylation map of mouse and human brain identifies target genes in Alzheimer’s disease. Brain.

[82] Yu L., Chibnik L.B., Srivastava G.P., Pochet N., Yang J., Xu J., Kozubek J., Obholzer N., Leurgans S.E., Schneider J.A. (2015). Association of Brain DNA methylation in SORL1, ABCA7, HLA-DRB5, SLC24A4, and BIN1 with pathological diagnosis of Alzheimer disease. JAMA Neurol..

[83] Nicolia V., Cavallaro R.A., López-González I., Maccarrone M., Scarpa S., Ferrer I., Fuso A. (2017). DNA Methylation Profiles of Selected Pro-Inflammatory Cytokines in Alzheimer Disease. J. Neuropathol. Exp. Neurol..

[84] Santana D.A., Smith M.A.C., Chen E.S. (2023). Histone Modifications in Alzheimer’s Disease. Genes.

[85] McCartney D.L., Stevenson A.J., Walker R.M., Gibson J., Morris S.W., Campbell A., Murray A.D., Whalley H.C., Porteous D.J., McIntosh A.M. (2018). Investigating the relationship between DNA methylation age acceleration and risk factors for Alzheimer’s disease. Alzheimer’s Dement..

[86] Oblak L., van der Zaag J., Higgins-Chen A.T., Levine M.E., Boks M.P. (2021). A systematic review of biological, social and environmental factors associated with epigenetic clock acceleration. Ageing Res. Rev..

[87] Grodstein F., Lemos B., Yu L., Klein H.U., Iatrou A., Buchman A.S., Shireby G.L., Mill J., Schneider J.A., De Jager P.L. (2021). The association of epigenetic clocks in brain tissue with brain pathologies and common aging phenotypes. Neurobiol. Dis..

[88] Griñán-Ferré C., Sarroca S., Ivanova A., Puigoriol-Illamola D., Aguado F., Camins A., Sanfeliu C., Pallàs M. (2016). Epigenetic mechanisms underlying cognitive impairment and Alzheimer disease hallmarks in 5XFAD mice. Aging.

[89] Tomita H., Vawter M.P., Walsh D.M., Evans S.J., Choudary P.V., Li J., Overman K.M., Atz M.E., Myers R.M., Jones E.G. (2004). Effect of agonal and postmortem factors on gene expression profile: Quality control in microarray analyses of postmortem human brain. Biol. Psychiatry.

[90] Hébert S.S., Horré K., Nicolaï L., Papadopoulou A.S., Mandemakers W., Silahtaroglu A.N., Kauppinen S., Delacourte A., De Strooper B. (2008). Loss of microRNA cluster miR-29a/b-1 in sporadic Alzheimer’s disease correlates with increased BACE1/beta-secretase expression. Proc. Natl. Acad. Sci. USA.

[91] Grasso M., Piscopo P., Confaloni A., Denti M.A. (2014). Circulating miRNAs as biomarkers for neurodegenerative disorders. Molecules.

[92] Geekiyanage H., Jicha G.A., Nelson P.T., Chan C. (2012). Blood serum miRNA: Non-invasive biomarkers for Alzheimer’s disease. Exp. Neurol..

[93] Satoh J., Kino Y., Niida S. (2015). MicroRNA-Seq Data Analysis Pipeline to Identify Blood Biomarkers for Alzheimer’s Disease from Public Data. Biomark. Insights.

[94] Xie B., Zhou H., Zhang R., Song M., Yu L., Wang L., Liu Z., Zhang Q., Cui D., Wang X. (2015). Serum miR-206 and miR-132 as Potential Circulating Biomarkers for Mild Cognitive Impairment. J. Alzheimer’s Dis..

[95] Burgos K., Malenica I., Metpally R., Courtright A., Rakela B., Beach T., Shill H., Adler C., Sabbagh M., Villa S. (2014). Profiles of extracellular miRNA in cerebrospinal fluid and serum from patients with Alzheimer’s and Parkinson’s diseases correlate with disease status and features of pathology. PLoS ONE.

[96] Bhatnagar S., Chertkow H., Schipper H.M., Yuan Z., Shetty V., Jenkins S., Jones T., Wang E. (2014). Increased microRNA-34c abundance in Alzheimer’s disease circulating blood plasma. Front. Mol. Neurosci..

[97] Cortini F., Roma F., Villa C. (2019). Emerging roles of long non-coding RNAs in the pathogenesis of Alzheimer’s disease. Ageing Res. Rev..

[98] Feng L., Liao Y.T., He J.C., Xie C.L., Chen S.Y., Fan H.H., Su Z.P., Wang Z. (2018). Plasma long non-coding RNA BACE1 as a novel biomarker for diagnosis of Alzheimer disease. BMC Neurol..

[99] Fotuhi S.N., Khalaj-Kondori M., Hoseinpour Feizi M.A., Talebi M. (2019). Long Non-coding RNA BACE1-AS May Serve as an Alzheimer’s Disease Blood-Based Biomarker. J. Mol. Neurosci..

[100] Souza V.C., Morais G.S., Henriques A.D., Machado-Silva W., Perez D.I.V., Brito C.J., Camargos E.F., Moraes C.F., Nóbrega O.T. (2020). Whole-Blood Levels of MicroRNA-9 Are Decreased in Patients with Late-Onset Alzheimer Disease. Am. J. Alzheimer’s Dis. Other Dement..

[101] Gong G., An F., Wang Y., Bian M., Yu L.J., Wei C. (2017). miR-15b represses BACE1 expression in sporadic Alzheimer’s disease. Oncotarget.

[102] Zhong Z., Yuan K., Tong X., Hu J., Song Z., Zhang G., Fang X., Zhang W. (2018). MiR-16 attenuates β-amyloid-induced neurotoxicity through targeting β-site amyloid precursor protein-cleaving enzyme 1 in an Alzheimer’s disease cell model. Neuroreport.

[103] Barbagallo C., Mostile G., Baglieri G., Giunta F., Luca A., Raciti L., Zappia M., Purrello M., Ragusa M., Nicoletti A. (2020). Specific Signatures of Serum miRNAs as Potential Biomarkers to Discriminate Clinically Similar Neurodegenerative and Vascular-Related Diseases. Cell. Mol. Neurobiol..

[104] Guo R., Fan G., Zhang J., Wu C., Du Y., Ye H., Li Z., Wang L., Zhang Z., Zhang L. (2017). A 9-microRNA Signature in Serum Serves as a Noninvasive Biomarker in Early Diagnosis of Alzheimer’s Disease. J. Alzheimer’s Dis..

[105] Lucci C., Mesquita-Ribeiro R., Rathbone A., Dajas-Bailador F. (2020). Spatiotemporal regulation of GSK3β levels by miRNA-26a controls axon development in cortical neurons. Development.

[106] Denk J., Boelmans K., Siegismund C., Lassner D., Arlt S., Jahn H. (2015). MicroRNA Profiling of CSF Reveals Potential Biomarkers to Detect Alzheimer`s Disease. PLoS ONE.

[107] Absalon S., Kochanek D.M., Raghavan V., Krichevsky A.M. (2013). MiR-26b, upregulated in Alzheimer’s disease, activates cell cycle entry, tau-phosphorylation, and apoptosis in postmitotic neurons. J. Neurosci..

[108] Leidinger P., Backes C., Deutscher S., Schmitt K., Mueller S.C., Frese K., Haas J., Ruprecht K., Paul F., Stähler C. (2013). A blood based 12-miRNA signature of Alzheimer disease patients. Genome Biol..

[109] Wang J., Chen C., Zhang Y. (2020). An investigation of microRNA-103 and microRNA-107 as potential blood-based biomarkers for disease risk and progression of Alzheimer’s disease. J. Clin. Lab. Anal..

[110] Yılmaz Ş.G., Erdal M.E., Özge A.A., Sungur M.A. (2016). Can Peripheral MicroRNA Expression Data Serve as Epigenomic (Upstream) Biomarkers of Alzheimer’s Disease?. Omics.

[111] An F., Gong G., Wang Y., Bian M., Yu L., Wei C. (2017). MiR-124 acts as a target for Alzheimer’s disease by regulating BACE1. Oncotarget.

[112] Tan L., Yu J.T., Liu Q.Y., Tan M.S., Zhang W., Hu N., Wang Y.L., Sun L., Jiang T. (2014). Circulating miR-125b as a biomarker of Alzheimer’s disease. J. Neurol. Sci..

[113] Kiko T., Nakagawa K., Tsuduki T., Furukawa K., Arai H., Miyazawa T. (2014). MicroRNAs in plasma and cerebrospinal fluid as potential markers for Alzheimer’s disease. J. Alzheimer’s Dis..

[114] Ansari A., Maffioletti E., Milanesi E., Marizzoni M., Frisoni G.B., Blin O., Richardson J.C., Bordet R., Forloni G., Gennarelli M. (2019). miR-146a and miR-181a are involved in the progression of mild cognitive impairment to Alzheimer’s disease. Neurobiol. Aging.

[115] Lee S.T., Chu K., Jung K.H., Kim J.H., Huh J.Y., Yoon H., Park D.K., Lim J.Y., Kim J.M., Jeon D. (2012). miR-206 regulates brain-derived neurotrophic factor in Alzheimer disease model. Ann. Neurol..

[116] Kenny A., McArdle H., Calero M., Rabano A., Madden S.F., Adamson K., Forster R., Spain E., Prehn J.H.M., Henshall D.C. (2019). Elevated Plasma microRNA-206 Levels Predict Cognitive Decline and Progression to Dementia from Mild Cognitive Impairment. Biomolecules.

[117] Hara N., Kikuchi M., Miyashita A., Hatsuta H., Saito Y., Kasuga K., Murayama S., Ikeuchi T., Kuwano R. (2017). Serum microRNA miR-501-3p as a potential biomarker related to the progression of Alzheimer’s disease. Acta Neuropathol. Commun..

[118] Bourdenx M., Martín-Segura A., Scrivo A., Rodriguez-Navarro J.A., Kaushik S., Tasset I., Diaz A., Storm N.J., Xin Q., Juste Y.R. (2021). Chaperone-mediated autophagy prevents collapse of the neuronal metastable proteome. Cell.

[119] Xu X., Sun Y., Cen X., Shan B., Zhao Q., Xie T., Wang Z., Hou T., Xue Y., Zhang M. (2021). Metformin activates chaperone-mediated autophagy and improves disease pathologies in an Alzheimer disease mouse model. Protein Cell.

[120] Sreenivasmurthy S.G., Iyaswamy A., Krishnamoorthi S., Reddi R.N., Kammala A.K., Vasudevan K., Senapati S., Zhu Z., Su C.F., Liu J. (2022). Bromo-protopine, a novel protopine derivative, alleviates tau pathology by activating chaperone-mediated autophagy for Alzheimer’s disease therapy. Front. Mol. Biosci..

[121] Lee Y.S., Lai D.M., Huang H.J., Lee-Chen G.J., Chang C.H., Hsieh-Li H.M., Lee G.C. (2021). Prebiotic Lactulose Ameliorates the Cognitive Deficit in Alzheimer’s Disease Mouse Model through Macroautophagy and Chaperone-Mediated Autophagy Pathways. J. Agric. Food Chem..

[122] Lu Y., Brommer B., Tian X., Krishnan A., Meer M., Wang C., Vera D.L., Zeng Q., Yu D., Bonkowski M.S. (2020). Reprogramming to recover youthful epigenetic information and restore vision. Nature.

[123] Nativio R., Donahue G., Berson A., Lan Y., Amlie-Wolf A., Tuzer F., Toledo J.B., Gosai S.J., Gregory B.D., Torres C. (2018). Dysregulation of the epigenetic landscape of normal aging in Alzheimer’s disease. Nat. Neurosci..

[124] Gräff J., Rei D., Guan J.S., Wang W.Y., Seo J., Hennig K.M., Nieland T.J., Fass D.M., Kao P.F., Kahn M. (2012). An epigenetic blockade of cognitive functions in the neurodegenerating brain. Nature.

[125] Yang S.S., Zhang R., Wang G., Zhang Y.F. (2017). The development prospection of HDAC inhibitors as a potential therapeutic direction in Alzheimer’s disease. Transl. Neurodegener..

[126] Sung Y.M., Lee T., Yoon H., DiBattista A.M., Song J.M., Sohn Y., Moffat E.I., Turner R.S., Jung M., Kim J. (2013). Mercaptoacetamide-based class II HDAC inhibitor lowers Aβ levels and improves learning and memory in a mouse model of Alzheimer’s disease. Exp. Neurol..

[127] Lozupone M., Dibello V., Sardone R., Castellana F., Zupo R., Lampignano L., Bortone I., Daniele A., Bellomo A., Solfrizzi V. (2023). The Impact of Apolipoprotein E (APOE) Epigenetics on Aging and Sporadic Alzheimer’s Disease. Biology.

[128] Qi L.S., Larson M.H., Gilbert L.A., Doudna J.A., Weissman J.S., Arkin A.P., Lim W.A. (2013). Repurposing CRISPR as an RNA-guided platform for sequence-specific control of gene expression. Cell.

[129] De Plano L.M., Calabrese G., Conoci S., Guglielmino S.P.P., Oddo S., Caccamo A. (2022). Applications of CRISPR-Cas9 in Alzheimer’s Disease and Related Disorders. Int. J. Mol. Sci..

[130] Park H., Hwang Y., Kim J. (2021). Transcriptional activation with Cas9 activator nanocomplexes rescues Alzheimer’s disease pathology. Biomaterials.

[131] Park H., Shin J., Kim Y., Saito T., Saido T.C., Kim J. (2022). CRISPR/dCas9-Dnmt3a-mediated targeted DNA methylation of APP rescues brain pathology in a mouse model of Alzheimer’s disease. Transl. Neurodegener..

